# Factors Affecting the Implementation, Use, and Adoption of Real-Time Location System Technology for Persons Living With Cognitive Disabilities in Long-term Care Homes: Systematic Review

**DOI:** 10.2196/22831

**Published:** 2021-01-20

**Authors:** Alisa Grigorovich, Yalinie Kulandaivelu, Kristine Newman, Andria Bianchi, Shehroz S Khan, Andrea Iaboni, Josephine McMurray

**Affiliations:** 1 KITE - Toronto Rehabilitation Institute University Health Network Toronto, ON Canada; 2 Institute for Health Policy, Management and Evaluation University of Toronto Toronto, ON Canada; 3 Daphne Cockwell School of Nursing Ryerson University Toronto, ON Canada; 4 Bioethics Program University Health Network Toronto, ON Canada; 5 Dalla Lana School of Public Health University of Toronto Toronto, ON Canada; 6 Department of Psychiatry University of Toronto Toronto, ON Canada; 7 Lazaridis School of Business & Economics Wilfred Laurier University Brantford, ON Canada

**Keywords:** assistive technology, real-time location system, long-term care, implementation science, dementia, Alzheimer disease, ambulatory monitoring, wearable technology, qualitative research

## Abstract

**Background:**

As the aging population continues to grow, the number of adults living with dementia or other cognitive disabilities in residential long-term care homes is expected to increase. Technologies such as real-time locating systems (RTLS) are being investigated for their potential to improve the health and safety of residents and the quality of care and efficiency of long-term care facilities.

**Objective:**

The aim of this study is to identify factors that affect the implementation, adoption, and use of RTLS for use with persons living with dementia or other cognitive disabilities in long-term care homes.

**Methods:**

We conducted a systematic review of the peer-reviewed English language literature indexed in MEDLINE, Embase, PsycINFO, and CINAHL from inception up to and including May 5, 2020. Search strategies included keywords and subject headings related to cognitive disability, residential long-term care settings, and RTLS. Study characteristics, methodologies, and data were extracted and analyzed using constant comparative techniques.

**Results:**

A total of 12 publications were included in the review. Most studies were conducted in the Netherlands (7/12, 58%) and used a descriptive qualitative study design. We identified 3 themes from our analysis of the studies: barriers to implementation, enablers of implementation, and agency and context. Barriers to implementation included lack of motivation for engagement; technology ecosystem and infrastructure challenges; and myths, stories, and shared understanding. Enablers of implementation included understanding local workflows, policies, and technologies; usability and user-centered design; communication with providers; and establishing policies, frameworks, governance, and evaluation. Agency and context were examined from the perspective of residents, family members, care providers, and the long-term care organizations.

**Conclusions:**

There is a striking lack of evidence to justify the use of RTLS to improve the lives of residents and care providers in long-term care settings. More research related to RTLS use with cognitively impaired residents is required; this research should include longitudinal evaluation of end-to-end implementations that are developed using scientific theory and rigorous analysis of the functionality, efficiency, and effectiveness of these systems. Future research is required on the ethics of monitoring residents using RTLS and its impact on the privacy of residents and health care workers.

## Introduction

### Background

In Canada, approximately 87% of people living in residential long-term care homes, or nursing homes, have dementia or another type of cognitive disability [1]. This percentage is expected to increase as the number of people older than 65 years is predicted to grow by 68% over the next 20 years [2] and the number of people living in North America with dementia is anticipated to increase by 63% [3]. The subsequent increase in the number of older adults living with cognitive disabilities is driving health care decision makers to consider the use of automation and novel technologies in an effort to decrease the cost of care while improving safety, quality of care, and efficiency in long-term care [4].

Real-time location systems (RTLS) consist of a software app and reference points that detect and synthesize positioning data from wireless tags or transmitters attached to objects or people. RTLS are being adopted in health care with the goal of improving health and safety. Uses of RTLS include monitoring handwashing [5], fall prevention [6], tracking of individuals [5,7] and assets [8], enhancing independence [9,10], and more recently for collecting health data [6,11-13]. RTLS use tags or badges attached to patients or assets, receiver devices, and software to continuously and inconspicuously monitor activity in real time over wireless networks.

The rising interest in the use of RTLS in long-term care homes is related not only to the ability to remotely track the movement of individuals and assets [5] but also to its potential to gather clinically significant objective streams of data to augment care providers’ subjective observation [6] and to increase safety and productivity in the face of an aging workforce [14,15]. RTLS are increasingly perceived as having the potential to enhance independence and improve the physical safety of residents living in long-term care while also reducing the widespread use of restrictive measures such as antipsychotic drugs, physical restraints, and secure units [16-18]. These *first-generation smart systems* [19] that use wireless geolocation have been successfully implemented in other industries such as insurance [20] and telecommunications [21] but present novel challenges when deployed in health care, particularly when they are also used to generate personal data from vulnerable people [22]. In fact, although the intent of care providers and organizations is to use RTLS to improve the lives of people living with dementia or other types of cognitive disabilities, their use may provide the opposite result by threatening residents' privacy and restricting their activity within the range of the technology [23].

Industry has leveraged the ability to collect, store, combine, and analyze large quantities of data about customers, despite data sets being anonymized. The disclosure of sensitive personal information, such as location data, poses reputational risk and risks to employability, insurability, and even civil liability. Moreover, the probability of harm increases with time and the frequency of data collection [24]. In health care, critical approaches to the implementation of monitoring technologies suggest the need for institutional accountability for any increased harm from their implementation, particularly for vulnerable populations [25]. Furthermore, there are recognized global standards around privacy for persons with disabilities, such as the United Nations Convention on the Rights of Persons with Disabilities, Article 22, that protects against privacy intrusions *regarding the place of residence or living arrangements* [26], which should also guide the implementation of monitoring technologies. Pursuant to recent criticism that the ethical standard of autonomy based on independence and noninterference is less relevant in long-term care [27], the introduction of RTLS into institutional settings requires a cautious approach that identifies all stakeholders’ perspectives (eg, residents, residents’ families, care providers, organizations, and society in general) and accounts for the benefits and risks to each that may result. Implementation of these technologies should only proceed when all risks have been accounted for and mitigated or when they are outweighed by agreed-upon aggregated benefits.

### Objectives

The question as to whether RTLS are valid, reliable, accurate, and adoptable in long-term care settings, particularly for the purpose of optimizing independence and safety for residents living with dementia or other cognitive disabilities is not well reported. In this paper, we explore the factors influencing the implementation, adoption, and use of such systems by conducting a systematic review to identify related literature from academic and peer-reviewed journals. The study of health care interventions, particularly focusing on the understanding and evaluation of their implementation, is important for researchers and health care managers. The interaction of agents such as organizations, care providers, and residents, their contexts, and their processes (both established and introduced by an intervention or novel technology) must be anticipated or, at the very least, accommodated in a successful implementation [28,29]. The authors are part of a large team of researchers exploring whether clinically relevant physical, cognitive, and mental well-being information about residents living with dementia can be derived from geolocation data. Before introducing RTLS software to various pilot locations that provide residential long-term care for adults with dementia or other cognitive disabilities, the authors seek to better understand the factors influencing the success of RTLS implementation, particularly in long-term care settings (eg, residential long-term care homes, long-stay psychiatric care facilities).

## Methods

We conducted a systematic review of the academic, peer-reviewed literature using the Joanna Briggs Institute (JBI) approach [30] developed to support evidence-based practice in health care settings [31]. The JBI methodology allows for the appraisal and integration of quantitative and qualitative forms of knowledge related to the research question so that findings from both methodologies may complement one another. This type of review strengthens the findings and generates more robust conclusions, thus making them more applicable to policy and practice [32]. Given the complexity and novelty of implementing and evaluating RTLS in long-term care settings, this methodology is especially suitable. Inclusion and exclusion criteria were prespecified, and procedural decisions were documented.

### Data Sources and Searching

In collaboration with a Medical Library Information Specialist, we searched the electronic databases Embase (1974: week 25, 2019), CINAHL (1981: August 3, 2019), MEDLINE (1946: August 3, 2019), and PsycINFO (1967: August 3, 2019). Search strategies included subject headings and keywords related to 3 concepts that were combined using *AND OR*. The search concepts were (1) illnesses, diagnoses, and disorders associated with cognitive disability; (2) long-term care settings (eg, long-term care homes); and (3) RTLS (eg, GPS, sensor-based systems). The Ovid MEDLINE search strategy is given in Multimedia Appendix 1. We restricted searches to adult populations (18 years and older), and primary research studies and reviews published in English. The hand-searching process included reviewing relevant journals and references of included studies and searching Google Scholar to identify unindexed references; selected papers were included in the screening process. An updated search was conducted on all databases, and citations of selected papers, to April 16, 2020, resulting in the addition of one paper.

### Study Selection

We included primary research studies and reviews that (1) used and/or described issues relating to RTLS, (2) focused on adults 18 years or older with cognitive disability, and (3) focused on long-term care homes or other types of residential facility settings. In the screening process, we excluded studies that (1) did not satisfy the inclusion criteria; (2) involved, or referred to, children or youth (17 years or younger); (3) focused on individuals without cognitive disabilities; or (4) were conference abstracts, editorials, and commentaries.

Before commencing the screening process, a calibration exercise was conducted to ensure reliability in correctly selecting paper for inclusion. This process entailed 4 researchers (YK, AG, JM, and AB) independently screening a random sample of the references. This process was repeated until unanimity was achieved; any conflicts were resolved through discussion. Titles and abstracts of all references were then screened independently for inclusion by at least 2 reviewers (YK and AG or JM). Conflicts were resolved via discussion or by a third reviewer. All references that were found to be eligible or potentially eligible underwent full-text screening to confirm eligibility, papers were screened independently by 3 reviewers (YK, AG, and JM), and conflicts were resolved by discussion.

### Data Extraction and Quality Assessment

Data and information from publications were systematically extracted using a prepiloted data extraction form created in Microsoft Excel. Extracted information included publication information and type, methodology, theoretical or conceptual frameworks used, target population characteristics, study participant characteristics, institution information, and nature of RTLS. One reviewer independently extracted this information (YK), and the extracted data were reviewed by at least 1 other reviewer (JM or AG). All included publications were critically appraised for quality using the corresponding JBI quality assessment tool for the publication type and/or study design [33]. One reviewer independently assessed quality, and assessments were confirmed by at least 1 other reviewer (JM or AG).

### Data Analysis

Selected publications were imported into NVivo 12.0 software (QSR International) for data management and analysis. An initial set of categories were identified a priori during the scope determination phase of the review; these provided the basis for the initial round of descriptive coding (eg, resident, outcomes, risk). We progressively developed new concepts, and codes were then added, updated, or deleted and then later combined into larger themes. By applying constant comparison techniques, we iteratively revised the coding framework and simultaneously coded and analyzed the data [34,35]. This began with 3 authors (JM, AG, YK) independently reading each paper several times to identify links between them and conducting an initial round of coding using the initial set of codes and identifying new ones (eg, open coding). We then discussed and further refined the coding framework [36]. This was followed by several rounds of axial coding and selective coding comparing and contrasting findings across studies, identifying patterns, and conceptualizing broader themes that were then iteratively discussed among the authors (JM, AG, YK), with conflicts resolved via consensus [37].

## Results

**Figure 1:**
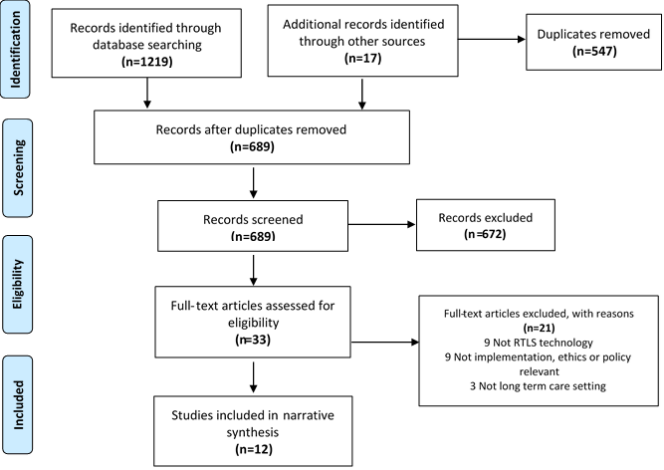
PRISMA (Preferred Reporting Items for Systematic Reviews and Meta-Analyses) flow diagram. RTLS: real-time locating system.

A total of 689 eligible references were identified through literature searches, and after screening, 12 publications were included in the review and are appended in Multimedia Appendix 2 [4,9,15,27,38-45]. Figure 1 shows a PRISMA (Preferred Reporting Items for Systematic Reviews and Meta-Analyses) flow diagram summarizing the results of the searching and screening process. All studies were published between 2010 and 2019. Most studies (7/12, 58%) were conducted in the Netherlands, and half of the total studies focused exclusively on persons living with dementia (6/12, 50%). A variety of study designs were used; 3 were quasi-experimental quantitative studies [38-40], 2 were concept mapping studies [41,42], 2 were qualitative case studies [4,43], 1 was a descriptive study [44], 1 was a qualitative descriptive study [9], 1 was an ethnographic study [45], and 2 were literature reviews (Niemeijer et al [45]; Oude Weernink et al [15]). Of the 12 publications, 2 were analyses of the same data set [4,43]. Of the publications examining the actual use, as opposed to the potential use, of RTLS (8/12, 67%), the most commonly used technologies were GPS and radio-frequency identification. Seven of the 12 publications collected qualitative data and/or feedback from individuals who would be tracked by the RTLS and other stakeholders interacting with the technology (eg, care providers); of these, 3 included data and feedback from residents. We conducted quality assessments of the included studies using the applicable JBI quality assessment tool. Quality assessments were performed independently by 1 review author (YK) and verified by a second review author (AG or JM). Most of the publications were considered good quality, except for the study by Bowen et al [44], which was rated as poor quality. None of the studies were excluded on the basis of quality assessments.

The selected studies were not overtly informed by implementation science theory [28]. Although normalization process theory [46] informed data collection for 1 study [43], its absence in the study design and analysis makes it difficult to extract meaning and better understand and control the barriers and facilitators that Greenhalgh et al [47] suggested actively affect the technology effectiveness. However, in our analysis of the collected data, we were able to identify 3 implementation-related themes (eg, barriers, enablers, agency and context) and several subthemes that may be useful for informing the execution of RTLS projects in residential care settings for adults living with cognitive disabilities.

### Theme 1: Barriers to Implementation

Implementation barriers are those factors that, if present, will actively impede the adoption and use of an RTLS. They are aggregated under 3 categories: lack of motivation for engagement; technology ecosystem and infrastructure; and myths, stories, and shared understanding. All are relevant to the resident, frontline care provider, and/or organizational contexts.

#### Lack of Motivation for Engagement

As with other technologies, an RTLS is likely to be used less often and at a fraction of its capabilities; lack of care providers’ and other stakeholders’ engagement is considered a barrier to technology adoption and meaningful use [48,49]. Hall et al [4] noted that the most common justification for the use of monitoring technologies such as RTLS for persons living with cognitive disability is to enhance safety and quality of care while also achieving institutional aims of functional efficacy, increased security (or reduction of risks), and *reduction of staff burden*, and if these expectations are not met, it elicited responses of disappointment and disengagement. Unless there is a compelling rationale for the need to increase a resident’s safety, frontline care providers appeared unwilling to support RTLS implementations [4]. Bowen et al [44] also found that care providers were less engaged when they viewed implementation as being conducted only for research purposes rather than for a permanent adoption that would affect their clinical routines. The persons who are the intended target for monitoring using RTLS in the selected papers are among the lowest adopters of novel technologies [50], consistent with Olphert and Damodaran’s [51] *digitally disengaged* populations, who experience cognitive and physical challenges with the uptake and sustained use of digital technologies. Although this may be a generational phenomenon, all of the studies indicated that resident participants in the selected studies had limited technological familiarity or expectations with respect to the RTLS (eg, study by Hall et al [43]) and were not perceived as being motivated or capable of understanding the implications of these technologies beyond their having more freedom of movement as a result [4]. However, they were able to discern others’ freedom as being different from their own as a result of others’ use of RTLS or find new private spaces using it themselves [27].

The direct and indirect costs of RTLS represent a distinct barrier to organizational engagement and implementation in long-term care settings where incremental technology costs are scrutinized carefully for cost efficiencies such as reduced staffing costs [40] and complexity versus high value outcomes such as greater safety in comparison with the status quo [4,43]. Furthermore, contractual agreements between institutions and incumbent suppliers may limit access to novel technologies and prevent personalization of digital solutions that improve adoption [43]. With economies of scale favoring larger institutional competitors, there is a higher likelihood of historic or contractual procurement obligations, preventing the entrance of new competitors willing to adapt to organizational needs [4].

Care providers’ and residents’ lack of understanding of organizational strategic goals and value of the RTLS and the data it produces may also negatively affect their engagement [4,9,44]. This might explain care providers removing or not replacing removable tags on patients, not responding to alerts [4,44], and not replacing tag batteries [4]. Providers are also reluctant adopters of the technology when one of the direct or indirect goals is workforce surveillance, whether it potentially enhances organizational reputation or quality of care [43]. Managers acknowledged the autocratic implications of RTLS on both care providers and residents, as did the RTLS developers, although one study reported a lack of awareness among providers and family members of residents when discussing these ethical tensions [4,43].

All selected papers, except Bowen et al [44], who focused on what they described as *critical adoption issues that must be overcome for deployment* (p 191), identified ethical concerns related to the use of RTLS. Hall et al [43] pointed to the complexity and discordance of the ethical issues that arise when the locations in which RTLS are installed have multiple *identities*—as a workspace, a space for care delivery, and as a residential domicile. Niemeijer et al [45] focused on the ethical concerns regarding surveillance technologies internationally but presented these issues not as *barriers* to adoption of RTLS per se, but rather considerations for an *ethical debate* (p 1129). The *debate* occurs when persons living with cognitive disabilities are perceived as lacking decision-making capacity and when providers’ and families’ duties to safeguard residents’ personal health and safety conflict with residents’ right to privacy and dignity [52]. For this reason, we do not present ethical dilemmas that result from the use of RTLS as a barrier but will explore them in detail in the *Discussion* section.

#### Technology Ecosystem and Infrastructure Challenges

Underperforming technology is one of the greatest barriers to successful RTLS implementation [15,53]. The ecosystem into which it is introduced must support its functionality. For instance, RTLS that take a walled-garden approach to protect an intellectual property or distinctiveness compromise interoperability with other institutional applications or platforms and add unnecessary and often unrealistic complexity that negates the value of their use [43]. Other issues include lack of range and signal strength [40], tag battery life [4,15,40], loss of antenna strength and connection [40], and tags that are easily soiled or misplaced [44]. The functionality of an RTLS can present direct and unintended barriers to adoption. For instance, the ability to remove a system’s wearable sensors is necessary for daily hygiene and maintenance purposes but presents an opportunity for these sensors to become lost—a catastrophic failure of this system, given its singular purpose to locate an object in time and space [44]. Some managers cited the technology’s lack of robustness and inability to withstand the rigors of daily use as reasons to reject or reconsider its use [4]. Facility design is often not ideal for RTLS installations, where thick walls or crowded space for installation of cables or new equipment might thwart their optimal functioning [44]. The frequency of false-positive alarms has been identified as 1 of the top 10 hazards in medical device technology [54] and a workflow disruptor that can lead to health care provider error [55] and fatigue [56]. Numerous studies mentioned the burden of frequent alarms generated by RTLS as negative for care providers and residents but conversely noted that they raised providers’ awareness of potentially risky incidents [4].

The lack of information in the papers related to location data storage, system security, and the specifics of data ownership and use was notable. The review paper by Oude Weernink et al [15] is the only one that identified system security (rather than security related to the safety of the individual wearer) as a potential barrier to the acceptance of RTLS. As the decision to use and place pervasive monitoring devices is not made by residents, the sacrifice of privacy for independence is not consciously made by residents. Niemeijer et al [45] suggest that discussions about data security and attitudes toward monitoring technologies are more skeptical in Europe than North America, perhaps reflecting the timing and adoption of the General Data Protection Regulation in 2016 regulating institutions’ safe collection and transfer of individuals’ private data. However, this was not evident in the selected literature.

#### Myths, Stories, and Shared Understanding

Insufficient training and clear communication preimplementation may contribute to the perception that use of RTLS supports the normative *blame culture* that arises when accidents occur in long-term care facilities [4,43]. Early care providers’ and other stakeholders’ engagement and instruction that discusses not only the functionality of the technology but also its intended benefits and anticipated challenges might address myths and disinformation [44] or potential resistance to RTLS use [15], all of which create barriers to implementation. Care providers were particularly worried about the potential for RTLS to be used for workplace oversight, although management in these studies appeared reluctant to acknowledge its active use in the supervision of care providers [4,43]. Inevitably, installation of an RTLS will affect existing routines and work practices [4], although they present less of a barrier to implementation when they fit with the technology’s processes and functionality [44]. Although there are indications that RTLS may be useful in identifying traffic flow bottlenecks of providers or residents, helping to prevent medical errors, and reducing resident and operational risks [15], these systems are insufficient correctives to problematic workplace attitudes and cultural issues [43]. The myths that RTLS have no discernible negative effects manifested in a variety of areas such as reduced commitment to training and hasty implementations and are justified by its apparent simplicity [4].

### Theme 2: Implementation Enablers

Implementation enablers are those factors that, if present, will actively facilitate the adoption and use of an RTLS. They are classified into 4 categories: understanding local workflows, policies, and technologies; usability and user-centered design; communication with care providers; and establishing policies, frameworks, governance, and evaluation.

#### Understanding Local Workflows, Policies, and Technologies

Bowen et al [44] described lessons learned where researchers and/or those implementing RTLS should ensure that they are aware of local and site-specific workflows, policies, technologies, and layout to ensure that the RTLS can be implemented effectively. Examples include ensuring that RTLS do not emit signals that might interfere with existing medical equipment and selecting RTLS wearables that are distinct from other types of wearables for ease of identification. In addition, trialing the technology before its implementation is key to identify and modify the technology to ensure that it does not potentially conflict or interfere with the existing workflows, policies, and other types of technology.

#### Usability and User-Centered Design

Many of the studies, both experimental and descriptive, suggest that the usability and acceptability of RTLS would be enhanced through the active engagement of all stakeholders, including residents, families, and care providers in implementation planning [15,40,42-45]. Bowen et al [44] found that engaging care providers and residents regarding the proposed RTLS and its components allowed them to modify the wearable component so that it was perceived as being less identifiable as a surveillance device by integrating a timepiece into the wristband. Hall et al [4,43] and Masciadri et al [40] suggested that designing an RTLS to be as unobtrusive as possible is an enabler, as it will be less noticeable to monitored residents. Niemeijer et al [41,45] added that implementation of RTLS should be person-specific and tailored to meet individual residents’ needs and requirements and their families’ preferences. Similarly, Masciadri et al [40] suggested that implementation should be context-specific and tailored to the preferences of stakeholders (eg, higher numbers of sensors for elevators and entrances for safety).

#### Communication With Care Providers

Communicating with, and providing training to, care providers regularly is suggested as enabling acceptance, compliance, understanding, and effective usage of RTLS [4,15,41,44]. However, communications with care providers seldom occurred through, or in concert with, leadership or management teams. Often, research teams would establish direct relationships and lines of communication with individual care providers, which improved their acceptance and understanding. Bowen et al [44] found that communicating and obtaining approval with/from leadership and management teams did not ensure that study information was communicated to all care providers in the home and suggested that researchers should make separate efforts to inform and accommodate care providers across different shift schedules [4,44]. Bowen et al [44] further suggested that conducting regular visits to implementation sites can help to address emerging concerns with providers and identify the root causes of challenges. Communication with care providers, as well as their training, should also be tailored to ensure that the aims, benefits, and usage of the technology, how it fits in with the values and practices of the home, and its functional usage [4,15,41] are clear.

#### Establishing Policies, Frameworks, Governance, and Evaluation 

Ensuring care providers and organizations are aware of whose responsibility it is to maintain the technology and to whom they report any issues would increase their confidence [41,42]. Similarly, relevant laws and policies with respect to privacy protection should be clearly defined and articulated to all stakeholders [42]. Contingency and emergency planning should also be discussed so that all are aware of how any potential failings will be addressed and by whom, as technologies become embedded in usual care [42]. Regular and clear evaluation of the monitoring technology should occur, including evaluating the quality of life of residents who are being monitored, to help guide decision making regarding whether the technology should be used. This decision-making process should be clearly described and communicated to everyone involved [41,43,45].

### Theme 3: Agency and Context

The implementation of an RTLS in any setting is influenced by what people do (agentic contributions) and the relationship between agents (individuals and groups) and their contexts (spatial, organizational, and normative). In long-term care homes, this is made more complex by the tensions and opportunities for actions that arise as a result of the goals and values of different stakeholders or groups of agents (eg, residents, family members, care providers, and organizations), their relationship with each other, and their relative decision-making power with respect to the adoption and use of the RTLS. Despite this complexity, the collected papers rarely acknowledge these tensions, and for the most part, explore the motivations and actions of care providers and organizations in isolation, largely divorced from a consideration of the relationship between them, with others, or with the context. This is problematic, as research in other congregate settings for older adults on monitoring technologies demonstrates that the failure to account for the mismatch in priorities between stakeholders may lead to resistance and discontinuation [57].

#### Residents

Although residents living with dementia are identified as the primary agent on whose behalf RTLS are implemented in long-term care settings, they are not the people who are driving their implementation. Little research has been done on the perceptions and experiences of residents with this type of technology, and they are generally perceived as being passive recipients of care. Furthermore, although the main justification for the need for an RTLS is that it will improve the independence and safety of residents living with dementia (eg, increased freedom of movement, prevention of harm), almost no research has explored this in practice. The available research focuses on the use of restraints and demonstrates that there is no difference in positive affect between residents with high activity of daily living (ADL) dependency who are physically restrained and residents monitored with an RTLS [39] and suggests that the introduction of RTLS does not lead to a reduction in the use of physical restraints [9].

Residents are described as being generally accepting of the use of RTLS [44], perhaps because they do not notice the technology and/or understand exactly how it works or influences their movements or interactions with providers [4,44]. Their occasional resistance to these technologies is dismissed as being because of a lack of understanding, which is attributed to their cognitive disability or inadequate technological design, rather than being accepted as a valid expression of choice. For example, Bowen et al [44] reported that residents who objected to these technologies did so because they mistakenly believed that the sensors could videotape, record their conversations, or restrict their movements in real time. Similarly, Hall et al [4] and Bowen et al [44] reported that explaining the purpose of the technology to residents—monitoring their movements remotely to enhance freedom of movement—could lead to rejection of an RTLS if the residents were not already aware that their movements were restricted or monitored otherwise. Residents’ removal of tags or wearable components of RTLS was attributed to poor design esthetics and/or uncomfortable placement (eg, male residents who refused to wear a pink wearable or perceived being tagged with a bracelet as stigmatizing) rather their objection to their purpose [4,44].

#### Family Members

Available research suggests that family members are treated by direct care staff and organizations as key decision makers with respect to implementation of RTLS, regardless of whether they act as a substitute decision maker or have formal power of attorney [58]. Family members generally endorse the use of monitoring technologies for residents if they believe that it enhances residents’ physical safety by either supporting earlier detection of risk of injury (eg, fall prevention, altercation with another resident) or risk of neglect or abuse from staff [40,41,44,45]. They also prioritize the physical safety of residents over the potential risk that these technologies may pose to their privacy and/or autonomy, with some perceiving technological monitoring without the use of video cameras as being more privacy-protecting than continuous in-person observation [41,45].

#### Care Providers

Similar to family members, providers perceived RTLS as being valuable components of care that support their responsibility to safeguard residents, and most research focused on their use of these systems to enhance in-person monitoring [4,9,43]. A key cited benefit of RTLS, generally supported by providers, was the ability to locate residents or monitor their movement remotely in real time [15,40,44]. Care providers, however, were less enthusiastic about the use of RTLS by the organization to locate or to monitor their activities and expressed worry that these technologies might be used to sanction them. For the most part, researchers did not explore this topic in depth, with some suggesting that providers’ worries were unfounded and because of their lack of understanding of the purpose of implementing an RTLS (eg, Bowen et al [44] called it a *myth*). Lack of trust in the technology because of its nascent development and ethical implications of using it with vulnerable populations can result in disengagement by care providers and residents either through nonuse or by use that ignores the risks and may result in overconfidence in its capabilities [45].

#### Organization

The main stakeholders behind implementations of RTLS appear to be senior leadership or management [4,43], who perceive these technologies as enhancing organizational protection from risk and liability (eg, prevention of injury to residents, defense against allegations of negligence) or as leading to cost savings (eg, reduction in providers, monitoring provider performance) [4,40,42-45]. These stakeholders also suggested that continuous data collection using RTLS may be useful for mitigating family members’ potential concerns about residents or as protection against complaints and litigation regarding neglect or abuse [40,44]. However, no studies have explored whether these benefits are realized in practice or whether organizations use RTLS in this manner. Management does, however, use RTLS to monitor providers’ performance in both covert and overt ways and perceives this as permissible based on organizations’ duty of care to residents [4,43]. In general, management considered the risks of RTLS to be less of a barrier to adoption than the risks to the institutional reputation of not meeting their duty of care through adequate facilities, services, and *innovation through people* [43].

## Discussion

With the anticipated aging of the population in many western countries [59-61], there is increasing interest in the potential of using technologies to improve the health, safety, and quality of life in long-term care homes while reducing the cost of care delivery. Our review of the scientific literature related to the use of RTLS to monitor individuals living with dementia or other types of cognitive disability in residential care settings reveals scant evidence of implementation science theory to inform and optimize outcomes, with the exception of 1 study [4,43]. Implementation science advocates for a clear understanding and resolution of issues related to novel technologies (in their broadest sense, including artifacts such as hardware, software, processes, and policies) and the context and setting in which it is being applied [62,63].

Much of the justification for the use of RTLS or other types of surveillance technologies in long-term care focuses on its potential to enhance the quality of life and physical safety of residents with cognitive disabilities, in particular through the avoidance of segregation or physical restraints [42]. Te Boekhorst et al [39] and Zwijsen et al [9] found that care providers perceive the use of surveillance technology as an *intermediate measure* for use before physical restraints, and participants in a study by Niemeijer et al [42] reported that although surveillance technology could prevent some forms of *freedom restriction*, it too can impede movement. The limited available evidence does not demonstrate a reduction in the use of restraints in practice, although it is unclear whether this was because of the misapplication of these technologies. For example, although Te Boekhorst et al [39] did not find that RTLS enhanced the quality of life for residents, they suggested that the restricted movement of residents with high ADL dependency might negate any independence benefits ascribed to the use of RTLS. Similarly, Zwijsen et al [9] suggested that an RTLS was not seen as a replacement for restraints because although it supported ubiquitous monitoring, it did not prevent risk or replace the need for providers to respond to risky situations [4]. Despite the suggestion that RTLS might reduce or replace in-person care (which is often an expressed concern of providers), the proper use and monitoring of the technology can be equally time-consuming [45]. The flood of data that require monitoring and action was seen as a potential deterrent to its adoption, in particular because of the lack of expertise in transforming data into clinically useful information in practice [4]. Although there is some value in improving communications about the technology and processes, Bowen et al [43] demonstrated that this does not translate into improved trust in the technology or management or adherence to protocols. Trust was also challenged when the RTLS was unreliable, requiring workarounds and additional provider time [4].

Technologies are increasingly *integrated* with humans, from wearables such as RTLS to biomedical implants such as pacemakers. As Latour and Venn [64] have pointed out, this intermingling means that technologies influence the behaviors and processes of humans and their institutions and vice versa. The features of some technologies, such as ubiquitous surveillance, may, as a result, be perceived as ethically untenable and unalterable and, thus, may not be appropriate for use in some populations or settings. Although the focus of this study is not on the ethics of RTLS implementation per se, half of the selected papers in this study report ethical issues to a greater or lesser extent. The presence of ethical arguments acknowledges that the use of RTLS introduces tensions between the values, goals, and autonomy of different stakeholders (eg, providers or organizations and residents) with respect to the use of RTLS, particularly when they are used in the care of persons living with dementia or other types of cognitive disabilities in long-term care settings [9,45,65]. This population is considered vulnerable, and both providers and organizations have a duty of care to them, suggesting an asymmetry of dependency and dignity that must be acknowledged and resolved before implementation of surveillance technology. Niemeijer et al [41] suggest that there is an *inherent duality… rooted in the moral conflict between safety and freedom* (p 303), where autonomy is offered in return for surveillance using RTLS [15]. They further point out that older adults in particular may feel that they have to accept an RTLS and sacrifice personal privacy for independence in a type of digital quid pro quo. Furthermore, family members and substitute decision makers favor the use of RTLS to enhance the physical safety of residents and perceive the potential risks to privacy as less important [4,42,43]. Although there is limited research on the experiences of persons living with dementia with RTLS, it is concerning that the available research suggests that their refusal of RTLS may not be respected [4,44]; resolving this dilemma is an ethical imperative to avoid coercive practices and respect resident autonomy. This is supported by recent efforts to develop and implement new technologies by integrating the ethical values and priorities of stakeholders into design and development [66,67].

### Recommendations

Health care organizations are bound by workplace safety and data privacy laws and regulations that struggle to keep up with novel technologies [68]. The literature suggests that care providers and residents must rely on the culture and values of each individual organization to guide the adoption of RTLS. Our analysis suggests that there is interest among providers and organizations to better integrate nascent RTLS into work routines that improve safety and quality of care. However, there are numerous barriers to their effective use, including lack of engagement, trust, shared understanding of goals, and a reliable and appropriate RTLS that meets the needs of residents and care settings. A variety of factors to improve implementation of RTLS in the long-term care setting should be considered, including integration with local workflows, policies, and technologies and ensuring that care providers, residents, and families are involved, and if possible, leading that process [65,69]. Given the financial constraints in this sector, policy makers should consider the creation of a variety of incentives to encourage the use of technology implementation best practices, such as improved staff training and technology infrastructure development, to optimize outcomes and impact on care quality [70].

Decision makers must also be aware that daily interactions with care providers are often the primary source of social contact for many residents, and thus, the replacement of human contact with surveillance technology may unintentionally increase social isolation [45]. Where possible, institutions should select a flexible, interoperable RTLS that allows customization to suit individual residents, settings, and technological ecosystems [43], understanding that resident and operational needs change over time [27]. Technology developers need to be sensitive to the financial constraints that dominate this sector [43] and aim to build technologies that are elegant in their simplicity and easy to use and focus on solving issues at a lower total cost of ownership (eg, financial, infrastructure, effort, privacy) [40] to institutions, providers, and residents. Issues such as poor battery life, lost tags, and interoperability are solvable with current technology [15]. Researchers are well advised to devise RTLS research protocols and pilot studies that prompt all relevant stakeholders to engage meaningfully with the technology.

The ethical use of RTLS in long-term care settings is similar to other decisions made by providers and management, in that it requires an assessment of the potential benefits and harms, both direct and indirect, to all stakeholders involved. However, this is complicated, given that persons living with dementia or other cognitive disabilities are not assessing the possible risks and benefits independently. Many preferred to defer to family members and other proxies rather than support residents in making independent choices about the use of monitoring technology [4]. Although substitute decision makers are legally mandated to make choices on behalf of residents who lack capacity, this does not absolve them, researchers, and care providers from asking whether the use of an RTLS is ethical, whether it is something residents would want, and what constitutes an acceptable risk as a result of their implementation. It is clear that the use of monitoring technologies with individuals who are nonverbal or have cognitive disabilities, to address issues such as mobility and independence and improve predictive clinical diagnostic capabilities, has preceded our understanding of the rights, risks, and unintended consequences of their use in long-term care. A complete assessment of these issues is recommended before any implementation, drawing on a growing body of work exploring ethical design and implementation of technologies for vulnerable populations [71,72]. Furthermore, it is optimal to anticipate conflicts of interest, such as incidental monitoring of care providers, before they become barriers to a successful implementation [45], and to explore other mechanisms for workforce management [43]. Although this study focused primarily on monitoring residents with RTLS, care providers were also monitored either directly with tags or indirectly through the imputation of activity related to residents, such as the time it took them to respond to an alarm. Although RTLS may offer data to help assess and optimize clinical workflows, automatically open and lock doors, and improve operations [15], it also has the potential to reduce employee privacy and may negatively affect care provider recruitment and retention [43] in a sector that already struggles to find qualified staff [73].

### Limitations

Although the quality of selected papers was acceptable, the limited number of studies and their small sample sizes reflective of qualitative research methodologies and exploratory research suggest that generalization of our results and recommendations for future research should be limited to similar population samples and sectors. Furthermore, 3 of the studies that were included in this review [38-40] contributed a limited amount of data and information to the main research objective of examining factors influencing the implementation, adoption, and use of RTLS.

### Conclusions and Future Research

There is a striking lack of evidence to support the justification and implementation of RTLS to improve the quality of life of residents and work of care providers in long-term care settings. More research related to RTLS use with individuals with cognitive disabilities is required and should include longitudinal evaluations of end-to-end implementations that are theoretically informed and include rigorous analysis of functionality, efficiency, and effectiveness in improving outcomes that are important to all stakeholders involved [4,42]. Empirical studies that rigorously evaluate the practical utility and adoption of RTLS and their related processes into a controlled environment, the value of customization to the requirements of individual residents, and technology infrastructure versus one-size-fits-all adoptions are also required to advance our understanding of their utility.

The use of RTLS to support workflow efficiencies, manage person-to-person contact, and collect clinical data for use in diagnosis and therapeutics is largely unexplored and offers opportunities for future research and use. The workload and care provider capacity for real-time monitoring and data management and analysis for optimal use and outcomes must be made explicit and included in cost-benefit analyses that precede the purchase and adoption of these systems. Furthermore, training for the operation and use of RTLS will require incremental skills training and increased staffing levels, at least in the short term, in a sector where availability of resources and high workloads are already problematic issues. Finally, ethical considerations related to monitoring residents with RTLS, and also directly or indirectly their care providers, are acknowledged but not settled and require further empirical research.

## Appendix

Multimedia Appendix 1 Ovid MEDLINE search strategy.

Multimedia Appendix 2 A summary of selected studies.

## Abbreviations

ADL: activities of daily living
JBI: Joanna Briggs Institute
RTLS: real-time locating system

## References

[1] Policy changes and educational supports help spur a decrease in inappropriate use of antipsychotics and restraints Internet. Canadian Institute for Health Information.

[2] Dementia in Canada: Summary Internet. Canadian Institute for Health Information.

[3] Alzheimer’s Disease International, Bupa (2013). Current and future cost and prevalence of Alzheimer’s disease and other dementias. Alzheimer’s Disease International (ADI) and Bupa.

[4] Hall A, Wilson C, Stanmore E, Todd CA (2017). Implementing monitoring technologies in care homes for people with dementia: a qualitative exploration using Normalization Process Theory. Int J Nurs Stud.

[5] van Hoof J, Verboor J, Oude Weernink C, Sponselee A, Sturm J, Kazak J, Govers G, van Zaalen Y (2018). Real-time location systems for asset management in nursing homes: an explorative study of ethical aspects. Information.

[6] Bowen ME, Crenshaw J, Stanhope SJ (2018). Balance ability and cognitive impairment influence sustained walking in an assisted living facility. Arch Gerontol Geriatr.

[7] Bowen ME, Kearns W, Crenshaw JR, Stanhope SJ (2019). Using a real-time locating system to measure walking activity associated with wandering behaviors among institutionalized older adults. J Vis Exp.

[8] Bykova A (2011). Institutes of innovative development: their role in regional clusters. Ekon Ann.

[9] Zwijsen SA, Depla MF, Niemeijer AR, Francke AL, Hertogh CM (2012). Surveillance technology: an alternative to physical restraints? A qualitative study among professionals working in nursing homes for people with dementia. Int J Nurs Stud.

[10] Robinson L, Hutchings D, Corner L, Finch T, Hughes J, Brittain K, Bond J (2007). Balancing rights and risks: conflicting perspectives in the management of wandering in dementia. Health Risk Soc.

[11] Akl A, Taati B, Mihailidis A (2015). Autonomous unobtrusive detection of mild cognitive impairment in older adults. IEEE Trans Biomed Eng.

[12] Bowen ME, Rowe M (2016). Intraindividual changes in ambulation associated with falls in a population of vulnerable older adults in long-term care. Arch Phys Med Rehabil.

[13] Jansen C, Diegelmann M, Schnabel E, Wahl H, Hauer K (2017). Life-space and movement behavior in nursing home residents: results of a new sensor-based assessment and associated factors. BMC Geriatr.

[14] Boulos M, Berry G (2012). Real-time locating systems (RTLS) in healthcare: A condensed primer. International Journal of Health Geographics.

[15] Oude Weernink C, Felix E, Verkuijlen P, Dierick-van Daele A, Kazak J, van Hoof J (2018). Real-time location systems in nursing homes: state of the art and future applications. J Enabling Technol.

[16] Ryden MB, Feldt KS, Oh HL, Brand K, Warne M, Weber E, Nelson J, Gross C (1999). Relationships between aggressive behavior in cognitively impaired nursing home residents and use of restraints, psychoactive drugs, and secured units. Arch Psychiatr Nurs.

[17] (2018). Dementia in long-term care. Canadian Institute for Health Information.

[18] Lachance C, Wright MD (2019). Avoidance of Physical Restraint Use among Hospitalized Older Adults: A Review of Clinical Effectiveness and Guidelines.

[19] Sassone A, Grosso M, Poncino M, Macii E (2016). Smart electronic systems: an overview. Smart Systems Integration and Simulation.

[20] (2014). Insurance and Technology: Evolution and Revolution in a Digital World Internet. Morgan Stanley Research.

[21] Rost M, Cramer H, Belloni N, Holmquist L (2010). Geolocation in the mobile web browser. UbiComp '10 Adjunct.

[22] Wood W, Bennett AV, Basch E (2014). Emerging use of patient generated health data in clinical research. Mol Oncol.

[23] Bennett B, McDonald F, Beattie E, Carney T, Freckelton I, White B, Willmott L (2017). Assistive technologies for people with dementia: ethical considerations. Bulletin of the World Health Organization.

[24] Altman M, Wood A, O'Brien D, Gasser U (2018). Practical approaches to big data privacy over time. Int Data Priv Law.

[25] Grigorovich A, Kontos P (2020). Towards responsible implementation of monitoring technologies in institutional care. Gerontologist.

[26] (2016). Convention on the Rights of Persons with Disabilities (CRPD). United Nations.

[27] Niemeijer AR, Depla MF, Frederiks BJ, Hertogh CM (2015). The experiences of people with dementia and intellectual disabilities with surveillance technologies in residential care. Nurs Ethics.

[28] May CR, Johnson M, Finch T (2016). Implementation, context and complexity. Implement Sci.

[29] May C (2013). Towards a general theory of implementation. Implement Sci.

[30] Introduction to JBI Systematic reviews. Joanna Briggs Institute.

[31] Jordan Z, Lockwood C, Munn Z, Aromataris E (2019). The updated Joanna Briggs Institute Model of Evidence-Based Healthcare. Int J Evid Based Healthc.

[32] Östlund U, Kidd L, Wengström Y, Rowa-Dewar N (2011). Combining qualitative and quantitative research within mixed method research designs: a methodological review. Int J Nurs Stud.

[33] Joanna Briggs Institute JBI Critical Appraisal Checklist for Qualitative Research. JBI Manual for Evidence Synthesis.

[34] Maykut P, Morehouse R (1994). Beginning Qualitative Research: A philosophic Practice Guide.

[35] Parry K (2004). Constant Comparison. The SAGE Encyclopedia of Social Science Research Methods.

[36] Lockyer S (2004). Coding qualitative data. The SAGE Encyclopedia of Social Science Research Methods.

[37] Corbin J, Strauss A (1998). Basics of Qualitative Research: Techniques and Procedures for Developing Grounded Theory.

[38] Yayama S, Yamakawa M, Suto S, Greiner C, Shigenobu K, Makimoto K (2013). Discrepancy between subjective and objective assessments of wandering behaviours in dementia as measured by the Algase Wandering Scale and the Integrated Circuit tag monitoring system. Psychogeriatrics.

[39] te Boekhorst S, Depla MF, Francke AL, Twisk JW, Zwijsen SA, Hertogh CM (2012). Quality of life of nursing-home residents with dementia subject to surveillance technology versus physical restraints: an explorative study. Int J Geriatr Psychiatry.

[40] Masciadri A, Comai S, Salice F (2019). Wellness assessment of Alzheimer’s patients in an instrumented health-care facility. Sensors.

[41] Niemeijer AR, Frederiks BJ, Depla MF, Legemaate J, Eefsting JA, Hertogh CM (2011). The ideal application of surveillance technology in residential care for people with dementia. J Med Ethics.

[42] Niemeijer A, Frederiks B, Depla M, Eefsting J, Hertogh C (2013). The place of surveillance technology in residential care for people with intellectual disabilities: is there an ideal model of application. J Intellect Disabil Res.

[43] Hall A, Brown Wilson C, Stanmore E, Todd C (2019). Moving beyond 'safety' versus 'autonomy': a qualitative exploration of the ethics of using monitoring technologies in long-term dementia care. BMC Geriatr.

[44] Bowen ME, Wingrave CA, Klanchar A, Craighead J (2013). Tracking technology: lessons learned in two health care sites. Technol Health Care.

[45] Niemeijer AR, Frederiks BJ, Riphagen II, Legemaate J, Eefsting JA, Hertogh CM (2010). Ethical and practical concerns of surveillance technologies in residential care for people with dementia or intellectual disabilities: an overview of the literature. Int Psychogeriatr.

[46] May CR, Mair F, Finch T, MacFarlane A, Dowrick C, Treweek S, Rapley T, Ballini L, Ong BN, Rogers A, Murray E, Elwyn G, Légaré F, Gunn J, Montori VM (2009). Development of a theory of implementation and integration: Normalization Process Theory. Implement Sci.

[47] Greenhalgh T, Robert G, Macfarlane F, Bate P, Kyriakidou O (2004). Diffusion of innovations in service organizations: systematic review and recommendations. Milbank Q.

[48] Weeks DL, Keeney BJ, Evans PC, Moore QD, Conrad DA (2015). Provider perceptions of the electronic health record incentive programs: a survey of eligible professionals who have and have not attested to meaningful use. J Gen Intern Med.

[49] Heisey-Grove D, Danehy L, Consolazio M, Lynch K, Mostashari F (2014). A national study of challenges to electronic health record adoption and meaningful use. Med Care.

[50] (2017). UK: smartphone ownership by age from 2012-2017 Internet. Statista.

[51] Olphert W, Damodaran L (2013). Older people and digital disengagement: a fourth digital divide?. Gerontology.

[52] Plastow NA (2006). Is Big Brother watching you? Responding to tagging and tracking in dementia care. Br J Occup Ther.

[53] Fisher JA, Monahan T (2012). Evaluation of real-time location systems in their hospital contexts. Int J Med Inform.

[54] ECRI (2020). ECRI Top 10 Health Technology Hazards for 2020. J Radiol Nurs.

[55] Cvach M (2012). Monitor alarm fatigue: an integrative review. Biomed Instrum Technol.

[56] Schikhof Y, Mulder I, Choenni S (2010). Who will watch (over) me? Humane monitoring in dementia care. Int J Hum Comput Stud.

[57] Berridge C (2017). Active subjects of passive monitoring: responses to a passive monitoring system in low-income independent living. Ageing Soc.

[58] (2000). A Guide to the Substitute Decisions Act Internet. Ministry of the Attorney General.

[59] Peek ST, Luijkx KG, Rijnaard MD, Nieboer ME, van der Voort CS, Aarts S, van Hoof J, Vrijhoef HJ, Wouters EJ (2016). Older adults' reasons for using technology while aging in place. Gerontology.

[60] Bodenheimer T (2005). High and rising health care costs. Part 1: seeking an explanation. Ann Intern Med.

[61] Carstairs S, Keon W (2009). Canada’s Aging Population: Seizing the Opportunity. Special Senate Committee on Aging Final Report.

[62] Schoville RR, Titler MG (2015). Guiding healthcare technology implementation: a new integrated technology implementation model. Comput Inform Nurs.

[63] Ross J, Stevenson F, Lau R, Murray E (2016). Factors that influence the implementation of e-health: a systematic review of systematic reviews (an update). Implement Sci.

[64] Latour B, Venn C (2017). Morality and Technology. Sage Publications.

[65] Bourbonnais A, Rousseau J, Lalonde M, Meunier J, Lapierre N, Gagnon M (2019). Conditions and ethical challenges that could influence the implementation of technologies in nursing homes: A qualitative study. Int J Older People Nurs.

[66] Cummings ML (2006). Integrating ethics in design through the value-sensitive design approach. Sci Eng Ethics.

[67] Pacifico Silva H, Lehoux P, Miller FA, Denis J (2018). Introducing responsible innovation in health: a policy-oriented framework. Health Res Policy Syst.

[68] Maguire C The struggle to keep up with data privacy regulations. Law Journal Newsletters.

[69] Bourbonnais A, Ducharme F, Landreville P, Michaud C, Gauthier M, Lavallée MH (2020). An action research to optimize the well-being of older people in nursing homes: challenges and strategies for implementing a complex intervention. J Appl Gerontol.

[70] Ko M, Wagner L, Spetz J (2018). Nursing home implementation of health information technology: review of the literature finds inadequate investment in preparation, infrastructure, and training. Inquiry.

[71] Ienca M, Wangmo T, Jotterand F, Kressig RW, Elger B (2018). Ethical design of intelligent assistive technologies for dementia: a descriptive review. Sci Eng Ethics.

[72] Fields LM, Calvert JD (2015). Informed consent procedures with cognitively impaired patients: a review of ethics and best practices. Psychiatry Clin Neurosci.

[73] Stone L, Keller J (2020). Ontario relying on volunteers to help staffing shortage in long-term care as feds release new guidelines. Globe Mail Internet Toronto, Canada.

