# Supplementary material for: Factors Affecting the Implementation, Use, and Adoption of Real-Time Location System Technology for Persons Living With Cognitive Disabilities in Long-term Care Homes: Systematic Review
Source: J Med Internet Res. 2021 Jan 20;23(1):e22831. doi: 10.2196/22831 (PMC7857945; doi:10.2196/22831)
Supplement: Multimedia Appendix 1 [file jmir_v23i1e22831_app1.docx]

**Appendix 1 Ovid Medline Search Strategy**

| 1 | exp Dementia/ or exp Cognitive Dysfunction/ or exp Cognition Disorders/ or exp Alzheimer Disease/ or exp Frontotemporal Dementia/ or exp Dementia, Vascular/ or exp Dementia, Multi-Infarct/ or exp Brain Injuries/ or Delirium, Dementia, Amnestic, Cognitive Disorders/ or exp Mental Health/ or Wernicke Encephalopathy/ |
| --- | --- |
| 2 | Mental health.tw,kf. |
| 3 | exp SCHIZOPHRENIA/ |
| 4 | exp Paranoid Disorders/ |
| 5 | (schizo$ or hebephreni$ or oligophreni$ or psychotic$ or psychosis or psychoses).tw,kf. |
| 6 | ((chronic$ or sever$) adj2 mental$ adj2 (ill$ or disorder$)).tw,kf. |
| 7 | exp intellectual disability/ or exp learning disorders/ or mentally disabled persons/ |
| 8 | ((mental* or intellect* or development* or learning*) adj1 (deficien* or disabilit* or retard* or disorder* or impair*)).tw,kf. |
| 9 | (intellect* adj1 function*).tw,kf. |
| 10 | ((angelman* or bardet-biedl* or brachmann-de lange* or cat* cry* or cri-du-chat* or coffin-lowry* or coffin* or crying cat* or de lange*or down* or fra* or fragile x or happy puppet* or labhart-willi* or labhart-willi-prader-fanconi* or laurence-moon* or laurence-moon-bardet-biedl* or laurence-moon-biedl* or x or martin-bell* or prader-labhart-willi* or prader-willi* or rett* or royer* or rubinstein* orrubinstein-taybi* or willi-prader* or william* or beuren*) adj2 syndrome*).tw,kf. |
| 11 | congenital hypothyroidism.tw,kf. |
| 12 | fetal alcohol spectrum disorders/ |
| 13 | ((fetal or foetal) adj2 alcohol).tw,kf. |
| 14 | cretinism.tw,kf. |
| 15 | fetal iodine deficiency disorder.tw,kf. |
| 16 | foetal iodine deficiency disorder.tw,kf. |
| 17 | congenital myxedema.tw,kf. |
| 18 | congenital myxoedema.tw,kf. |
| 19 | rett* disorder.tw,kf. |
| 20 | danon disease.tw,kf. |
| 21 | antopol disease.tw,kf. |
| 22 | glycogen storage disease type 3.tw,kf. |
| 23 | glycogen storage disease 3.tw,kf. |
| 24 | glycogen storage disease type III.tw,kf. |
| 25 | glycogen storage disease III.tw,kf. |
| 26 | cerebrovascular disorders/ or exp basal ganglia cerebrovascular disease/ or exp brain ischemia/ or exp carotid artery diseases/ or exp cerebrovascular trauma/ or exp intracranial arterial diseases/ or exp intracranial arteriovenous malformations/ or exp intracranial hemorrhages/ or stroke/ or exp brain infarction/ or stroke, lacunar/ or vasospasm, intracranial/ or vertebral artery dissection/ or exp hypoxia, brain/ |
| 27 | (intracranial embolism and thrombosis).tw,kf. |
| 28 | (stroke$ or post stroke or poststroke or post-stroke or apoplex$ or cerebral vasc$ or cerebrovasc$ or cva or SAH).tw,kf. |
| 29 | ((brain or cerebr$ or cerebell$ or vertebrobasil$ or hemispher$ or intracran$ or intracerebral or infratentorial or supratentorial or middle cerebr$ or mca$ or anterior circulation or basilar artery or vertebral artery) adj5 (isch?emi$ or infarct$ or thrombo$ or emboli$ or occlus$ or hypoxi$)).tw,kf. |
| 30 | ((brain$ or cerebr$ or cerebell$ or intracerebral or intracran$ or parenchymal or intraparenchymal or intraventricular or infratentorial or supratentorial or basal ganli$ or putaminal or putamen or posterior fossa or hemisphere$ or subarachnoid) adj5 (h?emorrhag$ or h?ematoma$ or bleed$)).tw,kf. |
| 31 | exp hemiplegia/ or exp paresis/ or exp aphasia/ or exp gait disorders, neurologic/ |
| 32 | (hempar$ or hemipleg$ or paresis or paretic or aphasi$ or dysphasi$).tw,kf. |
| 33 | exp brain damage, chronic/ or brain injuries/ or exp brain concussion/ or exp brain hemorrhage, traumatic/ or brain injury, chronic/ or diffuse axonal injury/ |
| 34 | Craniocerebral trauma/ or exp head injuries, closed/ or exp intracranial hemorrhage, traumatic/ |
| 35 | exp brain abscess/ or exp central nervous system infections/ or exp encephalitis/ or exp meningitis/ |
| 36 | (encephalitis or meningitis or head injur$).tw,kf. |
| 37 | exp brain neoplasms/ |
| 38 | ((brain or cerebr$) adj5 (injur$ or hypoxi$ or damage$ or concussion or trauma$ or neoplasm$ or lesion$ or tumor$ or tumour$ or cancer$ or infection$)).tw,kf. |
| 39 | (brain injur* or dement* or alzheimer*).tw,kf. |
| 40 | (lewy* adj2 bod*).tw,kf. |
| 41 | ('normal pressure hydrocephalus' and 'shunt*').tw,kf. |
| 42 | 'benign senescent forgetfulness'.tw,kf. |
| 43 | (pick* adj2 disease).tw,kf. |
| 44 | (creutzfeldt or jcd or cjd).tw,kf. |
| 45 | (cerebr* adj2 deteriorat*).tw,kf. |
| 46 | (cerebral* adj2 insufficient*).tw,kf. |
| 47 | binswanger*.tw,kf. |
| 48 | korsako*.tw,kf. |
| 49 | ((cogniti* or neurocogniti* or intellectual) adj2 (impair* or dysfunction* or disorder* or declin*)).tw,kf. |
| 50 | or/1-49 |
| 51 | Long-Term Care/ or Residential Facilities/ or Nursing Homes/ or Homes for the Aged/ |
| 52 | ((senior or elderly) adj living).tw,kf. |
| 53 | home? for the aged.tw,kf. |
| 54 | home? for the elderly.tw,kf. |
| 55 | ((care or rest or convalescen* or retirement or senior or elderly) adj home?).tw,kf. |
| 56 | ((aged or convalescent or institutional or long-term or residential or long term) adj care).tw,kf. |
| 57 | nursing home*.tw,kf. |
| 58 | (nursing adj3 (facility or facilities or residence? or center? or centre?)).tw,kf. |
| 59 | ("old age facility" or "old age facilities").tw,kf. |
| 60 | Old age home?.tw,kf. |
| 61 | LTC.tw,kf. |
| 62 | Longterm care.tw,kf. |
| 63 | Long term care.tw,kf. |
| 64 | ((residential or assisted living or long-term care or nursing or care or convalescent or retirement or rest) adj facilit*).tw,kf. |
| 65 | (residence? adj2 ('assisted living' or convalescen* or retire???? or 'long stay' or longstay or 'long term')).tw,kf. |
| 66 | institutionalization.tw,kf. |
| 67 | or/51-66 |
| 68 | exp Geographic Information Systems/ or exp Monitoring, Physiologic/ or exp Monitoring, Ambulatory/ or exp Remote Sensing Technology/ or exp Wireless Technology/ or Wearable Electronic Devices/ |
| 69 | ((beacon or monitoring or geolocation or location or assistive or surveillance or tracking) adj2 technolog*).tw,kf. |
| 70 | (resident? adj2 (monitor* or track*)).tw,kf. |
| 71 | ((remote or ambulatory or wireless or digital) adj2 (monitor* or sens*)).tw,kf. |
| 72 | continuous surveillance.tw,kf. |
| 73 | (GPS or RTLS).tw,kf. |
| 74 | Real time location system*.tw,kf. |
| 75 | Global positioning system*.tw,kf. |
| 76 | or/68-75 |
| 77 | 50 and 67 and 76 |
| 78 | 77 not ((exp child/ or exp infant/ or exp adolescent/) not exp adults/) |
| 79 | limit 78 to english language |
