# Supplementary material for: Factors Affecting the Implementation, Use, and Adoption of Real-Time Location System Technology for Persons Living With Cognitive Disabilities in Long-term Care Homes: Systematic Review
Source: J Med Internet Res. 2021 Jan 20;23(1):e22831. doi: 10.2196/22831 (PMC7857945; doi:10.2196/22831)
Supplement: Multimedia Appendix 2 [file jmir_v23i1e22831_app2.docx]

**Appendix 2. Summary of Selected Studies**

| **Study and year** | **Country** | **Study design** | **Study objective** | **Sites/units** | **Study participants** | **Residents’ cognitive disability** | **RTLS^a^ category** |
| --- | --- | --- | --- | --- | --- | --- | --- |
| Niemeijer et al [45], 2010 | The Netherlands and Norway | Literature review | To explore the evidence on the ethical and practical acceptability of surveillance technologies in the residential care of people with dementia and/or intellectual disabilities | N/A^b^ | N/A | Dementia; intellectual disabilities | Surveillance technologies |
| Niemeijer et al [41], 2011 | The Netherlands | Concept mapping | To explore care professionals’ perspectives on surveillance technologies by examining their ideal application in residential care for people with dementia | N/A | Physicians providing care to older adults, psychologists, ethicists, registered nurses, and certified nurse assistants | Dementia | Surveillance technologies |
| Zwijsen et al [9], 2012 | The Netherlands | Qualitative study | To examine the perspectives of care professionals on the use of surveillance technology as an alternative to physical restraints for persons with dementia | 7 | Physicians, managers, certified nurse assistants, student nurses, a psychologist, and a nurse | Not reported | GPS chips |
| Bowen et al [44], 2013 | United States | Descriptive | To describe experiences relating to adoption and compliance in use of RTLS to track the movement of registered nurses, other health care providers, and patients in a long-term care residential facility | 2 | Registered nurses and residents | Memory impairment from TBI^c^ and at risk of falls | RFID^d^ tags |
| Niemeijer et al [42], 2013 | The Netherlands | Concept mapping | To examine what the ideal application of surveillance technologies in the residential care for people with intellectual disabilities entails | N/A | Intellectual disability physicians, developmental psychopathologists, ethicists (academics), personal coaches, and support workers (professional carers) | Intellectual disability | N/A |
| Te Boekhorst et al [39], 2013 | The Netherlands | Quasi-experimental | To examine the social, mood, and behavioral dimensions of the quality of life among residents monitored using surveillance technology compared with those of residents with physical restraints | 6 | Residents | Dementia | GPS chips |
| Yayama et al [38], 2013 | Japan | Quasi-experimental | To compare the measurement of provider-administered outcomes with objective temporal and spatial movement outcomes obtained from an IC^e^ tag monitoring system | 1 | Residents | Alzheimer disease, vascular dementia, frontotemporal dementia, dementia related to alcoholism, and dementia with Lewy bodies | IC tags |
| Niemeijer et al [27], 2015 | The Netherlands | Ethnography | To examine how residents experience surveillance technologies and, in turn, assess how surveillance technologies might influence the autonomy of people with dementia or intellectual disabilities | 2 | Residents; member of the board representing clients, night nurses, a nursing assistant, a physician providing care for older adults, team leaders, an occupational therapist, relatives of one of the clients, an ID night care manager, and ID physician, and a cluster manager | Dementia; intellectual disability | GPS tags |
| Hall et al [4], 2017 | England | Embedded multiple-case design | To examine facilitators of and barriers to the uptake of monitoring technologies into routine practice in residential care homes, ethical issues relating to their use, and stakeholders’ perceptions of the balance of benefits and challenges from using monitoring technologies | 3 | Providers, relatives, and residents | Dementia | RFID tags |
| Oude Weernink et al [15], 2018 | The Netherlands | Narrative review | To examine the potential use of RTLS in nursing homes | N/A | N/A | Dementia | Pervasive technologies and RTLS |
| Hall et al [43], 2019 | England | Embedded multiple-case design | To examine the extent to which remote monitoring of care home providers, and equality of access to technologies, influenced the use of monitoring technologies within routine practices and the resulting ethical implications | 3 | Providers, relatives, and residents | Dementia | RFID tags |
| Masciadri et al [40], 2019 | Italy | Not reported | To examine how location tracking technologies may estimate the physical and social well-being of patients with Alzheimer disease | 1 | Caregivers, providers, operators, facility manager, residents | Alzheimer disease | Proximity detection methodology |

^a^RTLS: real-time locating system.

^b^N/A: not available.

^c^TBI: traumatic brain injury.

^d^RFID: radio-frequency identification.

^e^IC: integrated circuit.

References

45. Niemeijer AR, Frederiks BJM, Riphagen II, Legemaate J, Eefsting JA, Hertogh CMPM. Ethical and practical concerns of surveillance technologies in residential care for people with dementia or intellectual disabilities: An overview of the literature. Int Psychogeriatrics 2010 Nov;22(7):1129–1142. PMID:20199699

41. Niemeijer AR, Frederiks BJM, Depla MFIA, Legemaate J, Eefsting JA, Hertogh CMPM. The ideal application of surveillance technology in residential care for people with dementia. J Med Ethics 2011 May;37(5):303–310. [doi: 10.1136/jme.2010.040774]

9. Zwijsen SA, Depla MFIA, Niemeijer AR, Francke AL, Hertogh CMPM. Surveillance technology: An alternative to physical restraints? A qualitative study among professionals working in nursing homes for people with dementia. Int J Nurs Stud 2012 Feb;49(2):212–219. [doi: 10.1016/j.ijnurstu.2011.09.002]

44. Bowen ME, Wingrave CA, Klanchar A, Craighead J. Tracking technology: Lessons learned in two health care sites. Technol Heal Care 2013;21(3):191–197. [doi: 10.3233/THC-130738]

42. Niemeijer A, Frederiks B, Depla M, Eefsting J, Hertogh C. The place of surveillance technology in residential care for people with intellectual disabilities: Is there an ideal model of application. J Intellect Disabil Res 2013 Mar;57(3):201–215. [doi: 10.1111/j.1365-2788.2011.01526.x]

39. Te Boekhorst S, Depla MFIA, Francke AL, Twisk JWR, Zwijsen SA, Hertogh CMPM. Quality of life of nursing-home residents with dementia subject to surveillance technology versus physical restraints: An explorative study. Int J Geriatr Psychiatry 2013 Apr;28(4):356–363. [doi: 10.1002/gps.3831]

38. Yayama S, Yamakawa M, Suto S, Greiner C, Shigenobu K, Makimoto K. Discrepancy between subjective and objective assessments of wandering behaviours in dementia as measured by the Algase Wandering Scale and the Integrated Circuit tag monitoring system. Psychogeriatrics 2013 Jun;13(2):80–87. [doi: 10.1111/psyg.12011]

27. Niemeijer AR, Depla MFIA, Frederiks BJM, Hertogh CMPM. The experiences of people with dementia and intellectual disabilities with surveillance technologies in residential care. Nurs Ethics 2015;22(3):307–320. [doi: 10.1177/0969733014533237]

4. Hall A, Wilson CB, Stanmore E, Todd C. Implementing monitoring technologies in care homes for people with dementia: A qualitative exploration using Normalization Process Theory. Int J Nurs Stud [Internet] United Kingdom: Elsevier Science; 2017 Jul 1;72:60–70. [doi: http://dx.doi.org/10.1016/j.ijnurstu.2017.04.008]

15. Oude Weernink CE, Felix E, Verkuijlen PJEM, Dierick-van Daele ATM, Kazak JK, van Hoof J. Real-time location systems in nursing homes: State of the art and future applications. J Enabling Technol Emerald Group Publishing Ltd.; 2018 Jun 18;12(2):45–56. [doi: 10.1108/JET-11-2017-0046]

43. Hall A, Brown Wilson C, Stanmore E, Todd C, Wilson CB, Stanmore E, Todd C. Moving beyond ‘ safety ’ versus ‘ autonomy ’: A qualitative exploration of the ethics of using monitoring technologies in long-term dementia care. BMC Geriatr BMC Geriatrics; 2019 May 24;19(145):1–13. [doi: 10.1186/s12877-019-1155-6]

40. Masciadri A, Comai S, Salice F. Wellness Assessment of Alzheimer’s Patients in an Instrumented Health-Care Facility. Sensors MDPI AG; 2019 Aug 22;19(17):3658. [doi: 10.3390/s19173658]
